# Supplementary material for: Non-metallic T2-MRI agents based on conjugated polymers
Source: Nat Commun. 2022 Apr 14;13:1994. doi: 10.1038/s41467-022-29569-x (PMC9010432; doi:10.1038/s41467-022-29569-x)
Supplement: Supplementary file 1 — Supplementary information [file 41467_2022_29569_MOESM1_ESM.pdf]

Supplementary information:

Non-metallic T_2-MRI agents based on conjugated polymers

Qinrui Lin[1, 2], Yuhong Yang[3]*, Zhengzhong Shao[1, 2, 4, 5]*

[1]State Key Laboratory of Molecular Engineering of Polymers, Fudan University, Shanghai, China.

[2]Laboratory of Advanced Materials, Fudan University, Shanghai, China.

[3]Research Center for Analysis and Measurement, Fudan University, Shanghai, China.

[4]Department of Macromolecular Science, Fudan University, Shanghai, China.

[5]Jiangsu Collaborative Innovation Center of Biomedical Functional Materials, Nanjing Normal University, China.

*e-mail: yuhongyang@fudan.edu.cn; zzshao@fudan.edu.cn

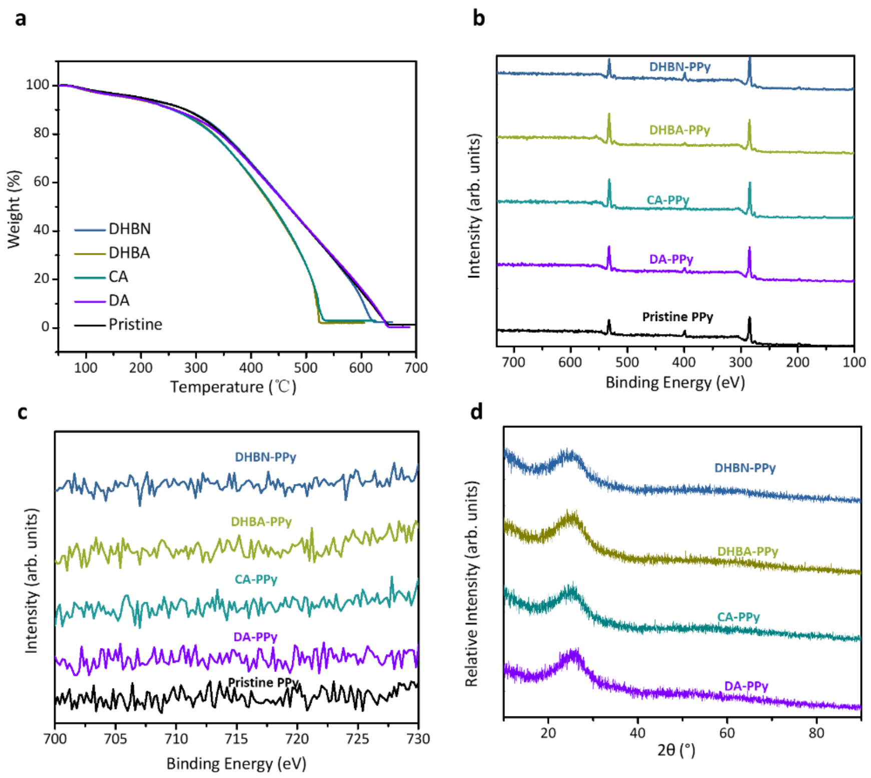

Fig. 1: Analyzing iron element in as synthesized PPy nanoparticles. a Thermalgravic analysis of pristine and catechols PPy nanoparticles. **b** Survey XPS spectra and **c** narrow scans of the Fe 2*p* region of the pristine and catechol derivatives. The Fe 2*p* peaks are not evident for all PPy nanoparticles. **d** XRD patterns of catechol PPy nanoparticles.

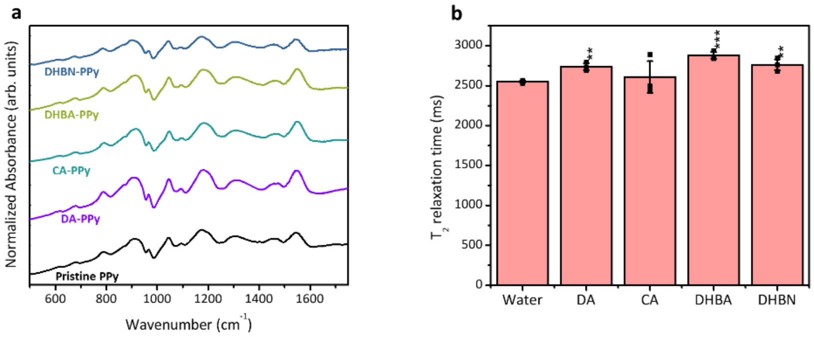

Fig. 2: Analyzing catechol derivatives in as synthesized PPy nanoparticles. a FTIR spectra of pristine PPy and catechols-PPys nanoparticles. **b** T_2 relaxation times of 1 mg/mL catechol derivatives in aqueous solutions. Error bars in b represent mean±s.d., with n=3 independent experiments. Statistical significance for comparison of water and catechol derivatives was determined by two-tailed Student's *t*-test (*$p < 0.05$, **$p < 0.01$ and ***$p < 0.001$). p_{DA}=2.26E-03, p_{CA}=0.643, p_{DHBA}=2.22E-04, p_{DHBN}=6.61E-03.

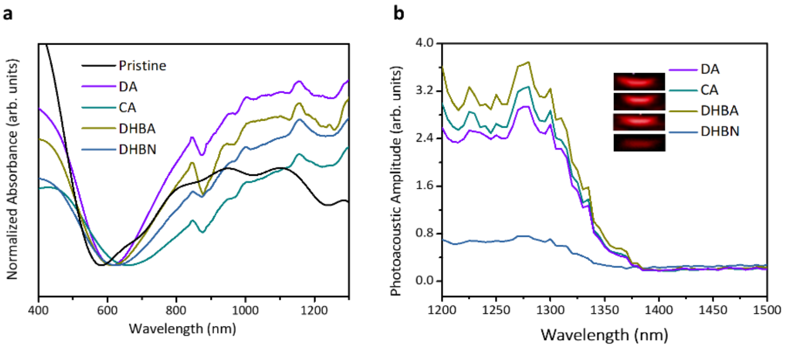

Fig. 3: Optical properties of catechol-PPy nanoparticles. (a) Absorption spectra and (b) photoacoustic Spectra of catechol-PPy nanoparticles. The inserted images are photoacoustic images acquired using 1 mg/mL nanoparticles.

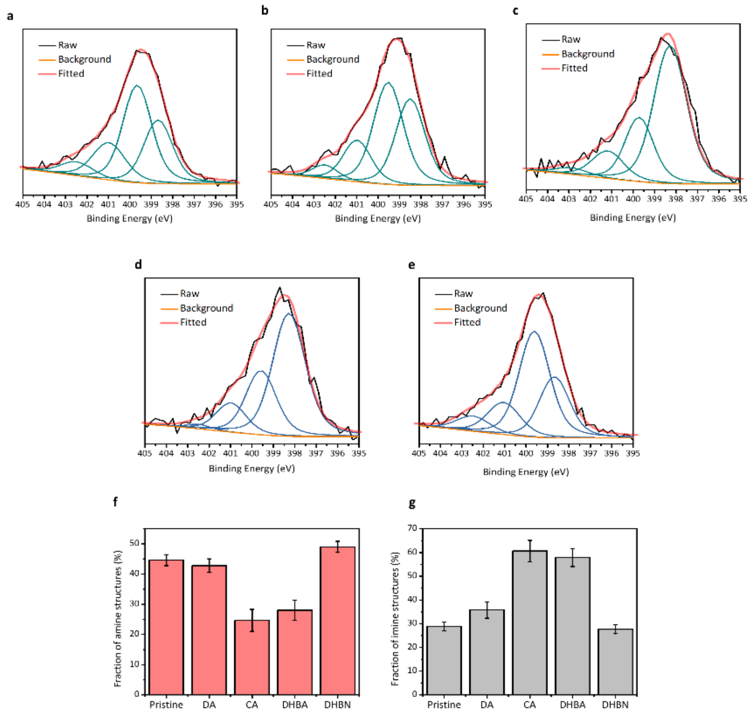

Fig. 4: Peak fitting analysis of XPS N 1*s* peak. a-e Peak fitting results of (a) pristine, (b) DA, (c) CA, (d) DHBA and (e) DHBN PPy nanoparticles. **f-g** Fractions of amine (f) and imine (g) structures estimated by peak fitting analysis. The error bars in f and g represent mean±s.d., with n=30 simulated spectra generated by Monte-Carlo simulations in CasaXPS software.

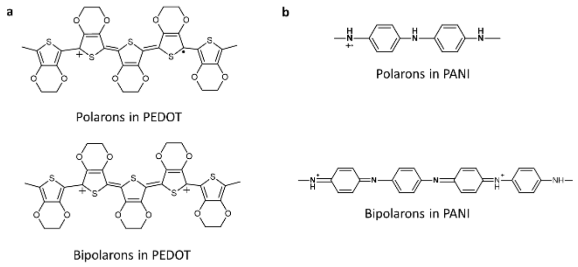

Fig. 5: Structures of polarons and bipolarons in PEDOT and PANI.

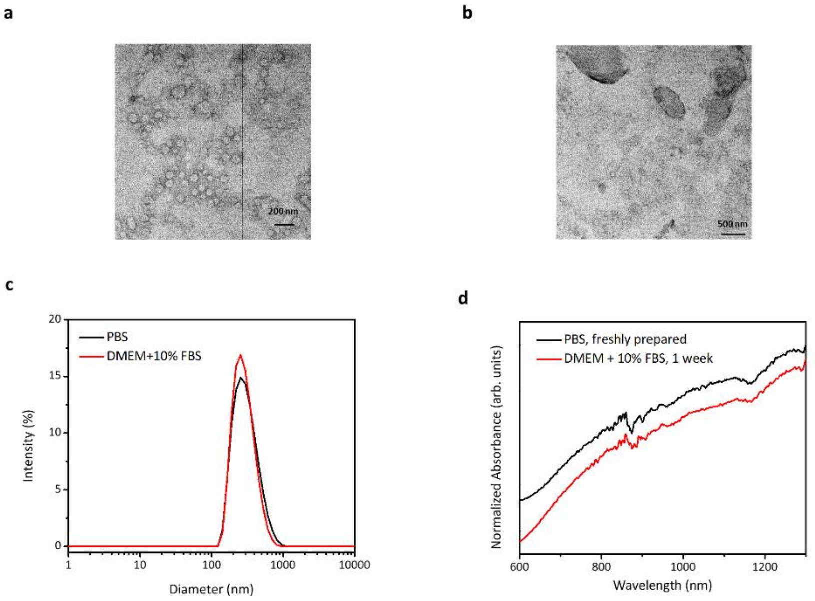

Fig. 6: Stability of DHBA-PPy@DSPE-PEG nanoparticles in physiological medium. a and **b** TEM images of DHBA-PPy@DSPE-PEG nanoparticles stained by phosphotungstic acid. Experiments were performed three times with similar results. (c) Size distributions and (d) UV-Vis-NIR absorption spectra of DHBA-PPy@DSPE-PEG nanoparticles in phosphate buffer (pH=7.4) and complete culture medium for 1 week at room temperature under sterilized environment.

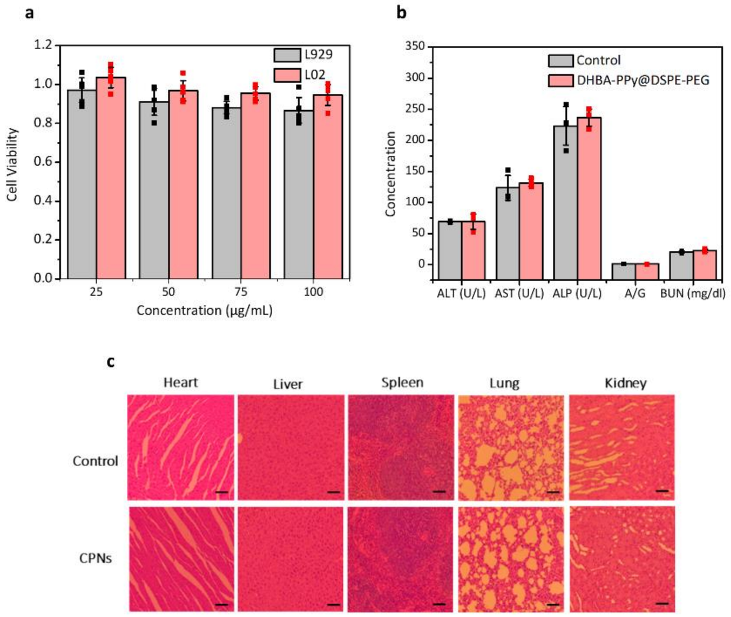

Fig. 7: In vitro and in vivo biosafety evaluation. a, Cell viabilities (assayed by CCK-8) of L929 and L02 cells incubated with DHBA-PPy@DSPE-PEG nanoparticles. Error bars in a represent mean±s.d., with n=5 wells of cells. **b,** Blood biochemistry indexes and **c,** Hematoxylin-eosin (H&E) images (The scale bar is 20 μm) of BALB/c mice injected with the nanoparticles and PBS (as control group) after 7 days. Error bars in b represent mean±s.d., with n=3 biologically independent animals.

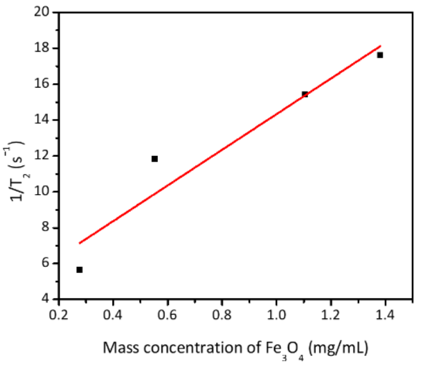

Fig. 8: The r_2 relaxivity of Fe_3O_4 nanoparticles is 9.9 mL/mg·s..

Table 1: Atomic concentrations calculated from the survey XPS spectra of different PPy nanoparticles. Besides C, N and O elements on the PPy chains, Cl is originated from the counter ions for polarons and bipolarons. And Si element is originate from the contamination in the environment like PDMS. The error analysis of atomic concentrations was performed using Monte-Carlo simulations in CasaXPS software. According to previous reports, Fe element in light element matrix is about 0.1%. Thus the existence of Fe element in PPy nanoparticles cannot be confirmed in the XPS spectra. The C/N ratio of different PPy nanoparticles varies between 7 and 12. The theoretical C/N ratio of PPy is 4 and that of DeTAB is 13. The C/N ratio of confirms the existence of residual surfactant DeTAB in the PPy nanoparticles.

	C	C-S.D.	N	N-S.D.	O	O-S.D.	Cl	Cl-S.D.	Fe	Fe-S.D.	Si	Si-S.D.	C/N
Pristine	74.75	1.06	8.70	0.93	13.35	0.60	2.77	0.30	0.04	0.08	0.39	0.56	8.6
DA	69.34	0.91	6.08	0.73	18.96	0.56	2.15	0.29	0.12	0.10	3.35	0.52	11.4
CA	70.32	0.97	6.08	0.79	18.47	0.61	1.69	0.28	0.12	0.07	3.32	0.49	11.6
DHBA	66.60	1.05	6.22	0.90	24.50	0.72	1.24	0.30	0.08	0.10	1.36	0.63	10.7
DHBN	71.25	0.99	10.01	0.80	13.35	0.53	2.20	0.34	0.08	0.09	3.11	0.55	7.1

Table. 2: Blood indexes of BALB/c mice injected with PBS (as control group) and DHBA-PPy@DSPE-PEG nanoparticles. Error bars represent mean±s.d., with n=3 biologically independent animals.

	Control	DHBA-PPy@DSPE-PEG
WBC (10^9/L)	5.38±0.31	5.20±0.54
RBC (10^{12}/L)	8.99±0.26	8.00±0.52
HGB (g/L)	125±5	111±9
HCT (%)	42.9±1.2	37.55±2.8
MCV (fL)	47.8±0.3	46.9±0.9
MCH (pg)	13.85±0.21	13.78±0.31
MCHC (g/L)	291±4	295±4
PLT (10^9/L)	683±49	542±80
MPV (fL)	4.73±0.13	4.9±0.2
