# Supplementary material for: Non-metallic T2-MRI agents based on conjugated polymers
Source: Nat Commun. 2022 Apr 14;13:1994. doi: 10.1038/s41467-022-29569-x (PMC9010432; doi:10.1038/s41467-022-29569-x)
Supplement: Supplementary file 3 — Reporting Summary [file 41467_2022_29569_MOESM3_ESM.pdf]

nature research

Corresponding author(s): Zhengzhong Shao and Yuhong Ynag

Last updated by author(s): Feb 25, 2022

# Reporting Summary

Nature Research wishes to improve the reproducibility of the work that we publish. This form provides structure for consistency and transparency in reporting. For further information on Nature Research policies, see our Editorial Policies and the Editorial Policy Checklist.

## Statistics

For all statistical analyses, confirm that the following items are present in the figure legend, table legend, main text, or Methods section.

| n/a | Confirmed | |
|---|---|---|
| ☐ | ☒ | The exact sample size (*n*) for each experimental group/condition, given as a discrete number and unit of measurement |
| ☐ | ☒ | A statement on whether measurements were taken from distinct samples or whether the same sample was measured repeatedly |
| ☐ | ☒ | The statistical test(s) used AND whether they are one- or two-sided<br>*Only common tests should be described solely by name; describe more complex techniques in the Methods section.* |
| ☒ | ☐ | A description of all covariates tested |
| ☒ | ☐ | A description of any assumptions or corrections, such as tests of normality and adjustment for multiple comparisons |
| ☐ | ☒ | A full description of the statistical parameters including central tendency (e.g. means) or other basic estimates (e.g. regression coefficient) AND variation (e.g. standard deviation) or associated estimates of uncertainty (e.g. confidence intervals) |
| ☐ | ☒ | For null hypothesis testing, the test statistic (e.g. *F*, *t*, *r*) with confidence intervals, effect sizes, degrees of freedom and *P* value noted<br>*Give P values as exact values whenever suitable.* |
| ☒ | ☐ | For Bayesian analysis, information on the choice of priors and Markov chain Monte Carlo settings |
| ☒ | ☐ | For hierarchical and complex designs, identification of the appropriate level for tests and full reporting of outcomes |
| ☒ | ☐ | Estimates of effect sizes (e.g. Cohen's *d*, Pearson's *r*), indicating how they were calculated |

*Our web collection on statistics for biologists contains articles on many of the points above.*

## Software and code

Policy information about availability of computer code

| | |
|---|---|
| Data collection | JobinYvon Horiba Standard XploRA Raman system was used to acquire Raman data. Bruker EPR spectrometer (E500) was used to acquire ESR data. Photoacoustic images were acquired using FujiFilm VisualSonics Photoacoustic Imaging System (Vevo LAZR-X). T2 relaxation times were acquired using a 0.5 T Niumag micro MR analyzing system (PQ001-20-015V). MRI images were acquired using a 0.5 T Niumag MesoMR magnetic resonance imaging system (MesoMR-60-H-I). XPS spectra were acquired using a Perkin-Elmer XPS System (PHI 5000C ESCA). Absorption spectra were aquired on a Perkin-Elmer spectrometer (Lambda 750). XRD measurements were performed using a Bruker diffractometer (D2 PHASER). The dynamic light scattering was performed on a Malvern particle size analyzer (ZS-90). FTIR spectra were acquired using a Thermofisher FTIR spectrometer (Nicolet 6700). TEM images were aquired on a FEI transmission electron microscope (Tecnai G2 20 TWIN). |
| Data analysis | Raman spectra were processed using Labspec (version 6) software. Polaron content was estimated by Xepr software (version 2.6) with spinfit function. Data analysis of absorption/FTIR/XRD spectra and statistical analysis were performed using Origin 8.5 and Microsoft Excel 2019. Molecular structures were performed with ChemDraw Professional 16.0. Photoacoustic images were analyzed using Fujifilm Vevo lab software version 3.1.0. The signal-to-noise ratio of ROI in the MRI images were analyzed using ImageJ software version 1.52a. Peak fitting analysis of XPS spectra was performed using CASAXPS version 2.3.24. |

For manuscripts utilizing custom algorithms or software that are central to the research but not yet described in published literature, software must be made available to editors and reviewers. We strongly encourage code deposition in a community repository (e.g. GitHub). See the Nature Research guidelines for submitting code & software for further information.

## Data

Policy information about availability of data

All manuscripts must include a data availability statement. This statement should provide the following information, where applicable:

- Accession codes, unique identifiers, or web links for publicly available datasets
- A list of figures that have associated raw data
- A description of any restrictions on data availability

All data supporting the findings from this study are provided in the paper and its supplementary information. All data are also available from the corresponding author upon reasonable request. Source data are provided with this paper.

# Field-specific reporting

Please select the one below that is the best fit for your research. If you are not sure, read the appropriate sections before making your selection.

☒ Life sciences ☐ Behavioural & social sciences ☐ Ecological, evolutionary & environmental sciences

For a reference copy of the document with all sections, see nature.com/documents/nr-reporting-summary-flat.pdf

# Life sciences study design

All studies must disclose on these points even when the disclosure is negative.

| | |
|---|---|
| Sample size | No sample size calculations were performed. The group sizes (n≥3 per group) represent the minimum number required for statistical analysis such as standard deviation and t-tests. For experiments on cell viabilities, the sample size (n=5) was increased due to the potential for increased variability in the number of seeded cells. |
| Data exclusions | No data was exclude from the analysis. |
| Replication | Experiments were repeated at least 3 independent times with similar results. All experiments were reproduced to reliably support conclusions stated in the manuscript. |
| Randomization | For in vivo imaging and blood biochemistry analysis, mice were randomly divided into experimental groups and each mouse was randomly assigned for injection of conjugated polymer nanoparticles and Fe3O4 nanoparticles. |
| Blinding | Hematoxylin&eosin staining images of major organs from mice injected with conjugated polymer nanoparticles and controls were acquired blinded. Blood assays were performed at the same time for all groups of a given experiment, so these studies were not blinded. In vitro experiments, in vivo imaging and data analysis were collected by instrument software without interference and processed with the same procedure. These data collection and analysis were carried out by the same investigators, so these studies were not blinded. |

# Reporting for specific materials, systems and methods

We require information from authors about some types of materials, experimental systems and methods used in many studies. Here, indicate whether each material, system or method listed is relevant to your study. If you are not sure if a list item applies to your research, read the appropriate section before selecting a response.

### Materials & experimental systems

| n/a | Involved in the study | |
|---|---|---|
| ☒ | ☐ | Antibodies |
| ☐ | ☒ | Eukaryotic cell lines |
| ☒ | ☐ | Palaeontology and archaeology |
| ☐ | ☒ | Animals and other organisms |
| ☒ | ☐ | Human research participants |
| ☒ | ☐ | Clinical data |
| ☒ | ☐ | Dual use research of concern |

### Methods

| n/a | Involved in the study | |
|---|---|---|
| ☒ | ☐ | ChIP-seq |
| ☒ | ☐ | Flow cytometry |
| ☒ | ☐ | MRI-based neuroimaging |

## Eukaryotic cell lines

Policy information about cell lines

| | |
|---|---|
| Cell line source(s) | L929 mouse fibroblasts and L02 human liver cells were purchased from from Stem Cell Bank, Chinese Academy of Sciences (Shanghai, China). |
| Authentication | All cell lines were authenticated by the supplier using Short Tandem Repeat test. |
| Mycoplasma contamination | No contamination was detected by the supplier. |
| Commonly misidentified lines (See ICLAC register) | These cell lines that we used were not listed in commonly misidentified lines in ICLAC Register |

## Animals and other organisms

Policy information about studies involving animals; ARRIVE guidelines recommended for reporting animal research

| | |
|---|---|
| Laboratory animals | Female BALB/c nude and BALB/c mice (6 weeks old) were obtained from the Shanghai SLRC Laboratory Animal Center (China). Housing conditions: temperature, 22~26 ℃; humidity, 40~60%; light,12 h on (7 a.m - 7 p.m.) and 12 h off. |
| Wild animals | This study did not involve wild animals. |
| Field-collected samples | This study did not involve field-collected samples. |
| Ethics oversight | All animal care and handling procedures were in agreement with the guidelines evaluated and approved by the ethics committee of Fudan University. |

Note that full information on the approval of the study protocol must also be provided in the manuscript.
